# Deficiency in Thrombopoietin Induction after Liver Surgery Is Associated with Postoperative Liver Dysfunction

**DOI:** 10.1371/journal.pone.0116985

**Published:** 2015-01-22

**Authors:** Stefanie Haegele, Florian Offensperger, David Pereyra, Elisabeth Lahner, Alice Assinger, Edith Fleischmann, Birgit Gruenberger, Thomas Gruenberger, Christine Brostjan, Patrick Starlinger

**Affiliations:** 1 Department of Surgery, Medical University of Vienna, General Hospital, Vienna, Austria; 2 Institute of Physiology, Medical University of Vienna, Vienna, Austria; 3 Department of Anesthesiology, Medical University of Vienna, General Hospital, Vienna, Austria; 4 Department of Internal Medicine, Brothers of Charity Hospital, Vienna, Austria; 5 Department of Surgery I, Rudolf Foundation Clinic, Vienna, Austria; ISMETT-UPMC Italy/ University of Catania, ITALY

## Abstract

**Background and Aims:**

Thrombopoietin (TPO) has been implicated in the process of liver regeneration and was found to correlate with hepatic function in patients with liver disease. With this investigation we aimed to determine if perioperative TPO levels were associated with postoperative outcome in patients undergoing liver resection.

**Methods:**

Perioperative TPO was analyzed prior to liver resection as well as on the first and fifth postoperative day in 46 colorectal cancer patients with liver metastasis (mCRC) as well as 23 hepatocellular carcinoma patients (HCC). Serum markers of liver function within the first postoperative week were used to define liver dysfunction.

**Results:**

While circulating TPO levels significantly increased one day after liver resection in patients without liver cirrhosis (mCRC) (P < 0.001), patients with underlying liver disease (HCC) failed to significantly induce TPO postoperatively. Accordingly, HCC patients had significantly lower TPO levels on POD1 and 5. Similarly, patients with major resections failed to increase circulating TPO levels. Perioperative dynamics of TPO were found to specifically predict liver dysfunction (AUC: 0.893, P < 0.001) after hepatectomy and remained an independent predictor upon multivariate analysis.

**Conclusions:**

We here demonstrate that perioperative TPO dynamics are associated with postoperative LD. Postoperative TPO levels were found to be lowest in high-risk patients (HCC patients undergoing major resection) but showed an independent predictive value. Thus, a dampened TPO increase after liver resection reflects a poor capacity for hepatic recovery and may help to identify patients who require close monitoring or intervention for potential complications.

## Introduction

Liver resection is considered the only curative treatment option for several neoplastic entities of the liver [1, 2]. The most significant factor determining postoperative morbidity and mortality is the ability of the remnant liver to regenerate [7]. Recently, platelets have been shown to play a pivotal role in liver regeneration after partial hepatectomy.

Thrombopoietin (TPO) is the main physiologic regulator of megakaryocyte maturation and platelet production [12]. Furthermore, TPO can act on platelet function and may therefore modify the effects of thrombocytes on liver regeneration [13, 14]. Besides its crucial role in platelet biology, TPO has also been shown to affect the liver directly. In particular, TPO was found to act as a growth factor for hepatic progenitor cells [15, 16]. Moreover, TPO has been reported to be expressed in liver endothelial cells, serving as an autocrine growth factor [17].

As hepatocytes are the major site of TPO production, TPO levels are partly decreased in patients with liver disease [18]. In line, those patients frequently suffer from perioperative thrombocytopenia. Aside from the fact that liver regeneration is impaired in patients with liver disease, the reduction in TPO and platelet counts seems to further reduce the capacity of hepatic recovery in these patients. Accordingly, preclinical models have demonstrated that TPO substitution is beneficial for postoperative liver regeneration, in particular in mice with liver cirrhosis [19, 20]. However, clinical evidence supporting the concept that TPO is of relevance in human liver regeneration is limited. We therefore aimed to determine the relation of perioperative TPO levels and postoperative liver dysfunction as a sign of impaired postoperative liver regeneration. As new orally active and highly potent thrombopoietin-receptor agonists are available, [21] this would support the concept that TPO substitution could be of benefit in high-risk patients undergoing liver resection.

## Patients and Methods

### Study Collective

A total of 69 patients undergoing liver resection were prospectively included in this study. To compare patients with and without liver disease only patients with metastatic colorectal cancer (N = 46) or hepatocellular carcinoma (N = 23) were enrolled. Baseline characteristics of patients including preoperative chemotherapy and portal venous embolization, surgical procedure, intraoperative Pringle maneuver and red blood cell use, as well as preoperative liver function were recorded and are listed and compared in Table 1. The type of resection was graded into major and minor resections according to the IHPBA Brisbane 2000 nomenclature (≤ 3 segments = minor, > 3 segments = major). [22] Routine blood sampling as well as plasma preparation were performed immediately prior to surgery (pre OP) and on day 1 (POD 1) as well as on day 5(POD 5) after liver resection. The Institutional Ethics Committee approved this study, which was performed according to the Declaration of Helsinki. (#424/2010, ethics committee medical university of Vienna, Borschkegasse 8b/E06, 1090 Vienna, ethik-kom@meduniwien.ac.at); all patients gave written informed consent. Furthermore, the study has been registered at the clinical trials registry (ClinicalTrials.gov Identifier: NCT01700231).

**Table 1:** Patient Demographics.

**Parameter**	**Collective(N = 69)**
Sex	
Male	50 (72%)
Female	19 (28%)
Neoplastic entity	
mCRC	46 (67%)
HCC	23 (33%)
Preoperative CTx	46 (67%)
Portal Venous Embolization	7 (10%)
Pringle maneuver	20 (30%)
RBC intraoperative (Units)	6 (8.7%)
Postoperative LD	
yes	10 (14.5%)
no	59 (85.5%)
Severe morbidity	14 (20%)
Hepatic resection	
Major	33 (47.8%)
Minor	36 (52.2%)
**Preoperative parameters**	**Median (Range)**
PDR %	20.8 (7.6–38.3)
R15%	4.5 (0.3–32.0)
B mg/dl	0.63 (0.22–3.17)
PT %	102 (40–145)
ALP U/l	84 (43–418)
GGT U/l	53 (11–699)
AST U/l	31 (17–208)
ALT U/l	32 (7–196)
Albumin g/l	42 (32.5–49.6)
Platelets (x10^3^/µl)	217 (92–470)
Age (years)	64 (22–85)
**Intraoperative parameters**	**Median (Range)**
Pringle maneuver min *	*14.5 (5–60)*
RBC intraoperative **	*2 (1–2)*
*Postoperative hospitalization*	*8 (4–90)*

ALT = alanine aminotransferase, ALP = alkaline phosphatase, AST = aspartate aminotransferase, CTx = chemotherapy, GGT = gamma-glutamyltransferase, HCC = hepatocellular carcinoma, LD = liver dysfunction, mCRC = metastatic colorectal cancer, PDR = plasma disappearance rate, PT = prothrombin time, RBC = red blood cells, R15 = retention rate after 15 min, SB = serum bilirubin.

* only patients with pringle included (20).

** only patients with intraoperative RBC included (6).

### Assessment of Preoperative Liver Function

Preoperative liver function was assessed by indocyanine green clearance test (ICG). ICG measurement was performed as previously described [23]. Briefly, pulse spectrophotometry was used to measure the blood ICG concentration. A dose of 25 mg ICG was dissolved in 20 ml of distilled water and was injected intravenously based on the body weight of patients. The plasma disappearance rate (PDR) and retention rate at 15 minutes (R15) were calculated automatically from the blood ICG concentration time course.

### Preoperative Portal Vein Embolization (PVE) and Surgical Technique

PVE was applied if major resection had to be performed, but the future remnant liver volume after liver resection was too small (< 30–25% of total liver volume) to allow for adequate postoperative liver function. Four weeks after PVE, hypertrophy of the non-embolized liver lobe was evaluated by computed tomography scanning. In case of inadequate hypertrophy of the future remnant liver, patients did not undergo surgery (which did not occur for any of the included patients).

All resections were performed or assisted by experienced hepatobiliary surgeons [24]. Initially, peritoneal carcinosis was excluded via a small laparotomy. Subsequently, the liver was mobilized and intraoperative ultrasound of the entire liver was performed. Dissection of hepatic parenchyma was performed using CUSA (Cavitron ultrasonic aspirator, Valleylab, Boulder, CO) and bipolar forceps. Intermittent Pringle maneuver was applied during parenchymal transection if necessary. A maximum of 15 minutes continuous portal inflow occlusion was always followed by a 5 minute period of reperfusion.

### Definition of Postoperative LD

Postoperative LD was evaluated based on the previously proposed criteria by Balzan et al [25]. The so-called “50–50 criteria” identify patients with a prothrombin time (PT) < 50% and a serum bilirubin (SB) level > 50 µmol/l corresponding to an SB concentration > 2.9 mg/dl. Balzan et al. were able to demonstrate that patients with an SB value > 50 µmol/l and a PT < 50% on POD 5 had a significant increase of postoperative mortality. Furthermore, in patients with significant morbidity this “50–50 criterium” was met 3 to 8 days before clinical evidence of complications. We thus recorded the respective liver function parameters during the first postoperative week. As the focus of this study was to detect delayed hepatic regeneration and not only complete liver failure, an SB concentration > 2.9 mg/dl and a PT value < 50% on any day within the first postoperative week were defined as postoperative LD.

### Optimized Plasma Preparation to Measure Circulating TPO Levels

An optimized plasma preparation technique was applied as previously described by us [26–29]. Briefly, blood was drawn into prechilled tubes containing citrate, theophylline, adenosine, and dipyridamole, it was immediately placed on ice and further processed within 30 min. After an initial centrifugation step at 1000 x g and 4°C for 10 min, the plasma supernatant was subjected to further centrifugation at 10000 x g and 4°C for 10 min (to remove remaining platelets). The supernatant was stored in aliquots at -80°C to avoid repeated cycles of freezing and thawing before analysis.

### Quantification of Blood Parameters

Plasma samples were analyzed by commercially available ELISA tests for human TPO (Quantikine; R&D Systems, Minneapolis, MN, USA) according to manufacturer’s instructions.

Perioperative parameters of liver function (SB, PT, ALT—alanine aminotransferase, AST—aspartate aminotransferase) were measured in serum samples by routine laboratory blood tests.

### Statistical Analysis

Statistical analyses were carried out with SPSS 20 Software (SPSS, Inc., Chicago, IL, USA) and were based on nonparametric tests (Mann–Whitney U test, Wilcoxon test, Spearman correlation). The Chi-squared test was used to evaluate frequencies between two groups. A receiver operating characteristic (ROC) analysis was performed to assess the potential of TPO levels to predict postoperative LD. In multivariate analysis (MVA) all parameters with a P value < 0.1 were included. Binary logistic regression analysis (method enter) was applied for calculations. Boxplot illustrations are given without outliers and extreme values to improve the resolution of interquartile ranges. P values < 0.05 were considered statistically significant.

## Results

### Plasma TPO Levels Increase After Liver Resection

**Figure 1:**
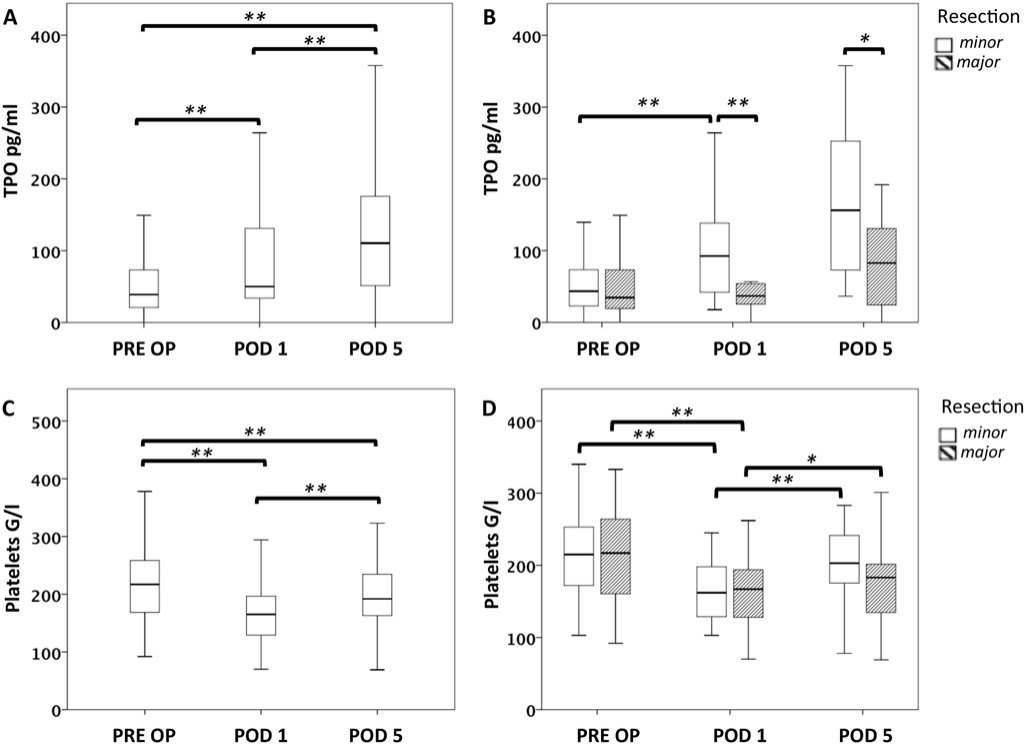
Perioperative fluctuations of platelet counts and circulating TPO in patients with major or minor resection. Plasma TPO levels were measured prior to surgery (PRE OP), on the first postoperative day (POD 1) and on POD 5. Circulating TPO levels are illustrated by boxplot in A. Patients were further divided in two groups undergoing major or minor liver resection (B). Comparably, platelet counts are illustrated for the entire collective (C) and for patients undergoing major or minor resection (D). * P < 0.05; ** P < 0.005.

As plasma TPO levels generally rise after platelet consumption, [30] we investigated the perioperative time course of TPO and platelets after liver resection (Fig. 1). As expected, we found a marked and significant increase in plasma TPO concentrations on POD 1 (median TPO PRE OP = 38.9 pg/ml, median TPO POD 1 = 50.1 pg/ml, P < 0.001) while circulating platelet counts dropped significantly (median platelet count PRE OP = 217 × 10^3^/ml, median platelet count POD 1 = 165 × 10^3^/ml, P < 0.001). However, while platelet counts started to recover again on POD 5, TPO plasma levels further increased (median TPO POD 5 = 110.5 pg/ml, PRE OP to POD 5: P < 0.001; POD 1 to POD 5: P = 0.001; median platelet count POD 5 = 192 × 10^3^/ml, PRE OP to POD 5: P = 0.002; POD 1 to POD 5: P < 0.001).

### Plasma TPO Levels Remain Low After Major Liver Resection

As the liver represents the major site of TPO production, we aimed to compare perioperative plasma TPO levels according to the extent of hepatic resection. Of note, patients undergoing major liver resection were found to exhibit substantially lower TPO plasma levels on POD 1 than patients with minor surgery (median TPO after minor resection = 92.4 pg/ml, median TPO after major resection = 36.8 pg/ml, P = 0.001). Furthermore, this difference in TPO expression was also observed on POD 5 (median TPO after minor resection = 156.2 pg/ml, median TPO after major resection = 82.7 pg/ml, P = 0.020). Nevertheless, platelet levels decreased to a comparable extent in patients with major and minor resections (median platelet count on POD 1 after minor resection = 162 × 10^3^/ml, median platelet count after major resection = 167 × 10^3^/ml, P = 0.812; median platelet count on POD 5 after minor resection = 203 × 10^3^/ml, median platelet count after major resection = 183 × 10^3^/ml, P = 0.205). Further characteristics of patients divided according to extent of resection are given in S1 Table.

### TPO Increase after Liver Resection is Impaired in HCC Patients

**Figure 2:**
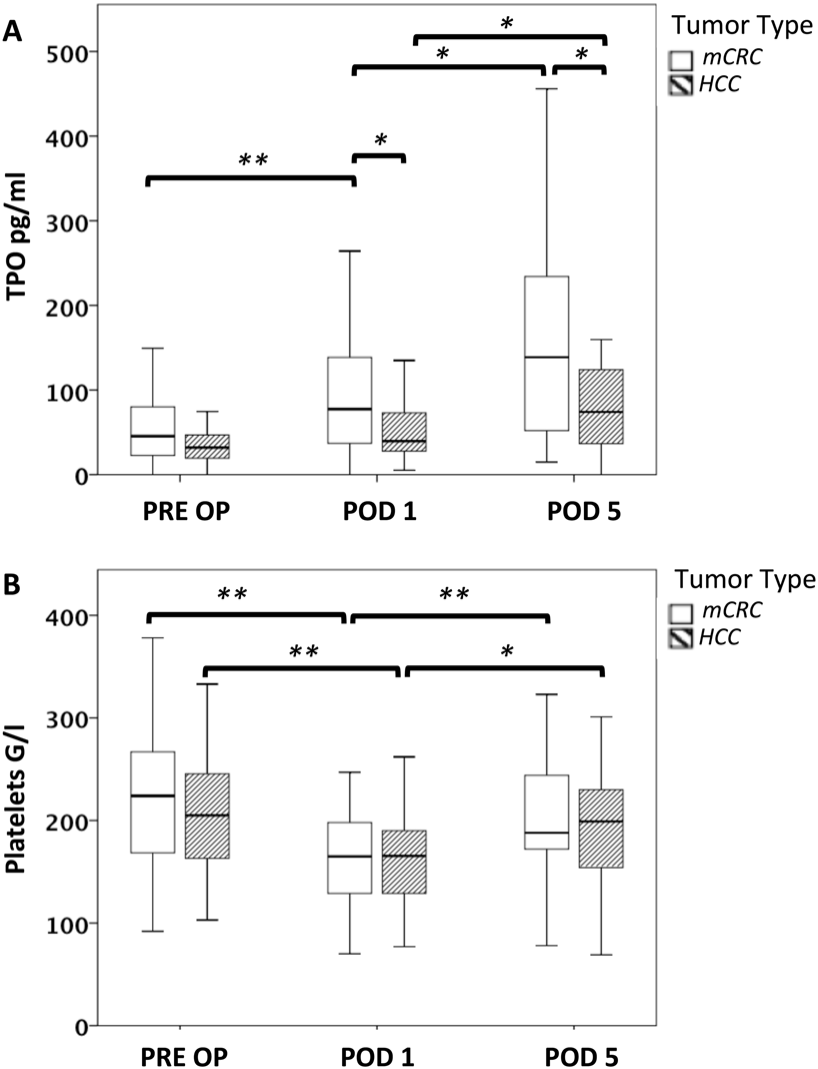
Perioperative changes in circulating platelet counts and TPO in patients with or without underlying liver disease. Plasma TPO levels were measured prior to surgery (PRE OP), on the first postoperative day (POD 1) and on POD 5. Patients were further divided in groups with underlying liver disease (HCC) or without underlying liver disease (mCRC). Circulating TPO levels are illustrated by boxplot in A. Comparably, platelet counts are illustrated in B. * P < 0.05; ** P < 0.005.

In line with previous reports showing that patients with liver cirrhosis have reduced circulating TPO levels, [18, 31–33] we found a moderate reduction in preoperative TPO concentrations in HCC as compared to mCRC patients. However, this difference did not reach statistical significance (median TPO mCRC = 45.4 pg/ml, median TPO HCC = 32.0 pg/ml, P = 0.111; Fig. 2A).

Importantly, these patients suffered from a reduced capacity to induce TPO expression after surgery: Plasma TPO levels failed to increase after liver resection in HCC patients (median TPO levels HCC POD 1 = 39.6 pg/ml, P = 0.085; Fig. 2A) and were found to be significantly lower than in mCRC patients (median TPO levels mCRC POD 1 = 77.38 pg/ml, P = 0.019; Fig. 2A). In contrast, TPO levels rose significantly in mCRC patients after surgery (P < 0.001). TPO levels in HCC patients showed a delayed increase on POD 5, but remained significantly lower than in mCRC patients (median TPO mCRC = 138.7 pg/ml, median TPO HCC = 74.0 pg/ml, P = 0.031; Fig. 2A). Accordingly, TPO levels were found to significantly increase in HCC and mCRC patients from POD 1 to POD 5 (mCRC: P = 0.014, HCC: P = 0.005; Fig. 2A). As for extent of resection, the tumor type had no effect on the perioperative fluctuations of platelet counts as shown in Fig. 2B.

### Absolute Values or Relative Increase in Postoperative TPO Levels are Associated with Postoperative LD

**Figure 3:**
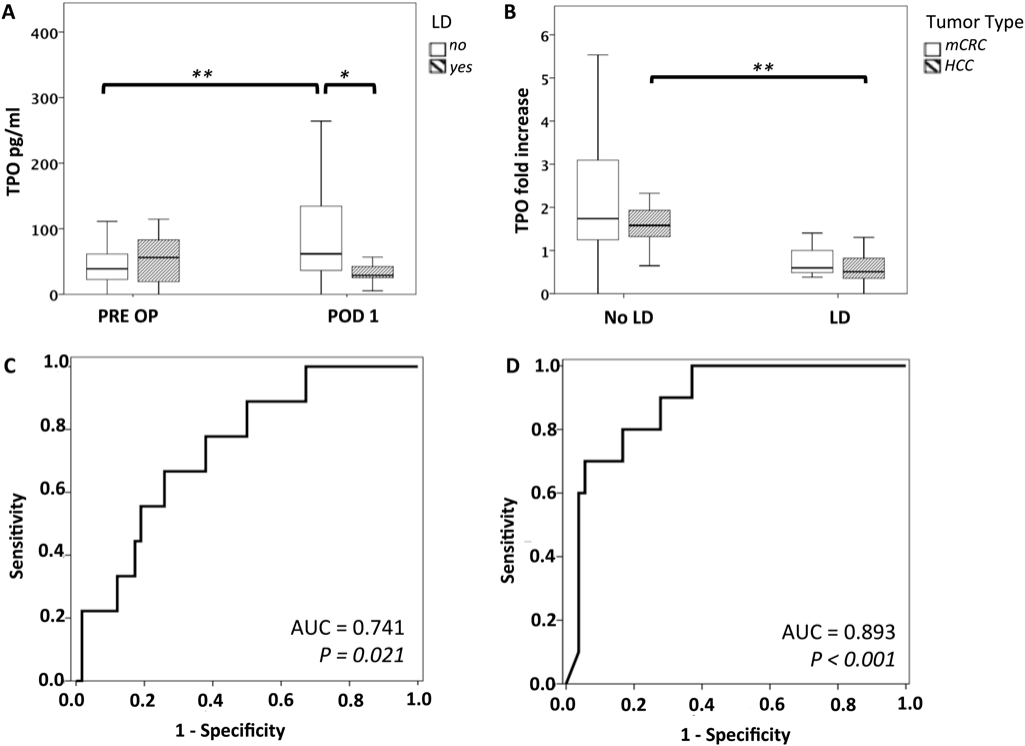
Perioperative TPO dynamics predict postoperative LD. Pre- and postoperative TPO levels are illustrated to demonstrate perioperative TPO changes in patients with or without postoperative LD (A). To reflect perioperative TPO dynamics the ratio of pre- to postoperative TPO levels was calculated (postoperative TPO: preoperative TPO). B illustrates fold increase in plasma TPO according to tumor type and liver dysfunction. Further ROC analysis was performed for LD and absolute levels of TPO on POD 1 (C) as well as relative TPO increase (D). * P < 0.05; ** P < 0.005.

Preclinical data suggest that perioperative TPO might affect postoperative liver regeneration [19]. Furthermore, TPO has been suggested to reflect liver function capacity [18, 31–33]. Therefore, we evaluated if patients with low perioperative plasma TPO levels would suffer from an increased incidence of postoperative LD. A total of 10 patients suffered from postoperative LD in our collective (basic characteristics of patients with or without postoperative LD are illustrated in S2 Table). While preoperative TPO levels did not differ significantly between patients with and without liver dysfunction, patients with postoperative LD were found to have substantially reduced TPO plasma levels on POD 1 (median no LD: 61.7 pg/ml, median LD: 28.9 pg/ml, P = 0.011; Fig. 3A). We further compared perioperative dynamics of TPO in patients with or without postoperative LD. A significant increase of TPO levels on POD 1 in relation to preoperative values was observed in patients who did not suffer from postoperative LD (median PRE OP: 38.9 pg/ml, median POD 1: 61.7 pg/ml, P < 0.001; Fig. 3A). However, patients who suffered from postoperative LD were unable to increase postoperative TPO expression (median PRE OP: 56.17 pg/ml, median POD 1: 28.9 pg/ml, P = 0.110; Fig. 3A). As perioperative TPO dynamics seemed to correlate with the risk of suffering from postoperative LD, the ratio (fold increase) from pre- to postoperative TPO levels was calculated. Accordingly, we found that patients without LD had a significantly higher (relative) increase in TPO than patients without postoperative LD (median LD: 0.513, median no LD: 1.64, P < 0.001). As we had observed a striking connection between the extent of resection and postoperative TPO levels, we reevaluated the association of TPO and liver dysfunction, including only major resections. Importantly, the “fold increase” in postoperative TPO was also reduced in the LD subgroup of patients with major liver resection (median LD: 0.506 median no LD: 1.34; P < 0.001). Furthermore, as we had observed that HCC patients were unable to induce TPO sufficiently after liver resection, we investigated whether the association of TPO increase on POD 1 with postoperative LD differed between tumor types. Remarkably, TPO values (postoperative fold increase) were lower in LD patients of both tumor types (borderline significant in mCRC patients). (HCC: median LD: 0.506 median no LD: 1.58, P < 0.001; mCRC: median LD: 0.596, median no LD: 1.74, P = 0.062, Fig. 3B). However, it should be noted that HCC patients had consistently lower TPO levels compared to mCRC patients.

To further characterize the relevance of perioperative TPO dynamics, we performed a ROC analysis. While absolute concentrations of plasma TPO on POD 1 were able to significantly predict postoperative LD with an AUC (area under the curve) value of 0.741 (P = 0.021, Fig. 3C), the postoperative fold increase in TPO was even more potent in predicting LD (AUC: 0.893; P < 0.001, Fig. 3D). When we further evaluated the incidence of postoperative LD in patients who were unable to increase circulating TPO levels after surgery (postoperative TPO level ≤ preoperative TPO level), we found a significant increase in postoperative LD from 5.7% to 54.5% compared to patients showing an increase in postoperative TPO levels after surgery (P < 0.001). Importantly, this association was also observed when only patients with major resections or HCC were analyzed (major resections: 11.1% vs. 60.0%, P = 0.006; HCC: 13.3% vs. 66.7%, P = 0.015).

Finally, patients with postoperative LD were found to suffer from poor clinical outcome as severe morbidity (grade III–V acc. to Dindo et al. [34]) was found to significantly increase from 15% to 50% in patients suffering from postoperative LD (P = 0.012). Furthermore, postoperative hospitalization was significantly prolonged from a median of 8 to 12 days in LD patients (P = 0.019).

### Postoperative TPO Increase is an Independent Predictor of Postoperative LD

Multivariate analysis (MVA) was further performed to assess the predictive value of TPO for postoperative LD. All recorded patient parameters showing an association with postoperative LD with a P-value < 0.1 in univariate analysis were included in the MVA (Table 2). As postoperative SB and PT values were used to define LD, these parameters were not comprised in uni- and multivariate tests. Upon univariate analysis the type of underlying carcinoma (tumor type), the type of resection, preoperative chemotherapy (CTx), preoperative gamma-glutamyltransferase (GGT), preoperative aspartate aminotransferase (AST), postoperative GGT, pringle/ischemia time in minutes and postoperative TPO increase predicted postoperative LD with a P-value < 0.1 and were therefore included in multivariate analysis. Upon MVA, however, only the postoperative rise in TPO was able to independently predict postoperative LD (P = 0.017, CI: 0.007–0.616).

**Table 2:** Predictors of LD after Hepatectomy.

	**Univariate Analysis**				**Multivariate Analysis**			
**Variables**	**B**	**Exp(B)**	**95% CI**	**P**	**B**	**Exp(B)**	**95% CI**	**P**
**Basic Characteristics**								
Age	0.023	1.024	0.967–1.083	0.421				
Sex	0.141	1.152	0.265–5.006	0.850				
Tumor type	-1.836	0.159	0.037–0.693	0.014	-1.200	0.301	0.002–56.67	0.653
Type of resection	-2.575	0.076	0.009–0.641	0.018	-0.846	0.429	0.027–6.903	0.551
CTx	1.836	6.271	1.443–27.253	0.014	1.435	4.200	0.023–764.7	0.589
PDR %	-0.106	0.900	0.791–1.024	0.108				
R15 %	0.058	1.060	0.949–1.184	0.299				
Portal venous embolization	0.019	1.019	0.109–9.493	0.987				
Pringle maneuver	-1.076	0.341	0.087–1.343	0.124				
Pringle/Ischemia time min	0.049	1.050	0.995–1.109	0.077	0.101	1.107	0.946–1.295	0.207
Intraoperative RBC amount	0.572	1.771	0.603–5.199	0.298				
Preoperative blood parameters								
SB	0.410	1.507	0.527–4.312	0.445				
PT	-0.027	0.974	0.942–1.006	0.112				
GGT U/l	0.006	1.006	1.000–1.011	0.051	0.006	1.006	0.980–1.032	0.662
AST U/l	0.019	1.019	1.001–1.036	0.036	-0.022	0.979	0.926–1.034	0.440
ALT U/l	0.012	1.012	0.995–1.031	0.173				
ALP U/l	0.004	1.004	0.994–1.014	0.415				
Albumin g/l	0.018	1.018	0.818–1.267	0.874				
Platelets G/l	-0.001	0.999	0.990–1.009	0.897				
**Postoperative blood parameters**								
**TPO increase**	**-2.455**	**0.086**	**0.018–0.408**	**0.002**	**-2.740**	**0.065**	**0.007–0.616**	**0.017**
GGT U/l	0.009	1.009	1.000–1.018	0.042	-0.004	0.996	0.957–1.037	0.860
AST U/l	0.000	1.000	0.998–1.002	0.736				
ALT U/l	-0.001	0.999	0.997–1.002	0.608				
ALP U/l	-0.001	0.999	0.981–1.018	0.940				
Albumin g/l	0.047	1.048	0.863–1.272	0.638				
Platelets G/l	-0.008	0.992	0.978–1.006	0.283				

ALT = alanine aminotransferase, ALP = alkaline phosphatase, AST = aspartate aminotransferase, CTx = chemotherapy, GGT = gamma-glutamyltransferase, PDR = plasma disappearance rate, PT = prothrombin time, R15 = retention rate after 15 min, RBC = red blood cell, SB = serum bilirubin, TPO = thrombopoietin.

## Discussion

Substantial technical improvements in the field of liver surgery have increased the possibility to extend the volume of resected parenchyma, leaving a very small remnant liver [4]. However, extensive liver resection bears the increased risk of postoperative LD. The occurrence of LD in turn increases the patient′s susceptibility to major complications, especially severe infections, which may result in early postoperative death [5, 6]. Accordingly, early postoperative detection of patients at risk to develop postoperative LD is of critical importance. Based on this study we present clinical evidence that perioperative TPO dynamics after liver resection represent a suitable predictor for postoperative outcome. In particular, we demonstrate that TPO levels significantly increase after liver resection only in patients without underlying liver cirrhosis (mCRC) or receiving minor resections, while patients with underlying liver disease (HCC) or undergoing major hepatectomy failed to induce TPO postoperatively. Importantly, a lack in postoperative TPO increase on POD1 after liver resection was found to specifically predict postoperative LD, which remained an independent predictor upon multivariate analysis. We further confirmed that postoperative LD resulted in poor clinical outcome regarding severe morbidity and hospitalization time.

TPO is a glycoprotein that is continuously synthesized by hepatocytes [35]. However, TPO blood levels are not necessarily decreased during chronic liver disease or acute liver failure [36, 37] but seem to decline in advanced states of liver fibrosis and cirrhosis, when low TPO concentrations are found to correlate with thrombocytopenia [38–40]. Since HCC typically develops in patients with liver cirrhosis, a lower TPO blood level in this patient population seemed feasible. While HCC patients had slightly reduced preoperative TPO levels compared to mCRC patients, this difference did not reach statistical significance. Of note, all included HCC patients were required to have an adequate remnant liver function (only Child A patients were resected). This excludes patients with extensive liver cirrhosis and might explain why HCC patients had only moderately reduced TPO levels. Furthermore, the inclusion of only Child A HCC patients might also explain why other parameters affected by liver disease, such as ICG-clearance or platelet counts and albumin were unable to predict postoperative LD.

However, while TPO levels significantly increased in mCRC patients after surgery, the postoperative TPO induction was markedly reduced in HCC patients. This suggests that patients with liver cirrhosis have an impaired ability to launch the postoperative TPO response. Comparably, also extent of surgery determined postoperative TPO levels, as patients undergoing a major resection failed to induce postoperative TPO expression. This indicates that after a stressor (after surgery), functional liver tissue might be the most relevant source of circulating TPO. It has previously been a matter of debate whether reduced TPO levels in patients with liver disease are indeed a result of the failing liver. Based on the observation that not only liver transplantation [31, 33] but also partial splenic embolization [41, 42] was able to restore circulating TPO levels and platelet numbers, an additional contribution by hypersplenism was suggested [43, 44]. With respect to TPO induction after hepatic surgery, our results point to a predominant role of the remaining liver. A potential stressor for the postoperative TPO increase might be IL-6, which is known to increase during liver resection [45] and is able to stimulate TPO production by hepatocytes [46]. The simple reduction of platelet counts, also a known mechanism of circulating TPO regulation, seems less likely, as it would not explain the differences in HCC and mCRC patients as well as in the extent of resection that we observed in our investigation.

The postoperative TPO increase was of major clinical relevance as it predicted postoperative LD (AUC 0.830) and remained an independent predictor in multivariate analysis. Importantly, deficiency in TPO induction on POD 1 was associated with LD when mCRC and HCC patients were analyzed separately and also when only major resections were included. This suggests that, although major resection and tumor type (underlying liver disease) are affecting perioperative TPO dynamics, a lack of postoperative TPO increase remains an unfavorable risk factor in all subgroups.

While TPO levels can be viewed as a result of the remaining functional liver mass, TPO in turn has been shown to promote liver regeneration in mice [19, 20]. TPO stimulates hepatic progenitor cell growth and acts as an autocrine growth factor for liver endothelial cells [16, 17]. Furthermore, as platelet derived growth factors are crucial in liver regeneration, TPO may promote hepatic regeneration indirectly by affecting platelet function [47]. In particular, TPO may induce platelet aggregation and prime platelets for stimulation with other agonists [14, 47]. Furthermore, TPO accelerates platelet adhesion and might therefore play a relevant role in platelet extravasation at the time of liver regeneration [13]. While there is a multitude of preclinical reports suggesting a functional involvement of TPO in hepatic regeneration, it should be emphasized that this clinical observational study was not designed to determine if TPO is causally related with postoperative liver regeneration or merely a reflection of the remaining hepatic capacity (or both).

Of note, no close association of perioperative platelet and TPO levels was observed. Previous reports in patients with liver transplantation have documented that platelet counts increase with a delay of 6–14 days after surgery in relation to TPO levels [31, 33]. Accordingly, the postoperative TPO induction might not exert its effects within the first week but rather later after surgery. As patients undergoing liver resection are usually discharged within the first postoperative week, the impact on platelet counts might not be apparent in our study. Furthermore, this may argue for the importance of direct effects by TPO on liver regeneration rather than indirect mechanisms affecting circulating platelet numbers.

In terms of treatment, TPO mimetics are approved and applied successfully as second line therapy for idiopathic thrombocytopenic purpura [48, 49]. In addition, TPO mimetics have also shown promising results in the treatment of patients with liver disease by efficiently increasing platelet counts prior to elective invasive procedures [50]. In this study we found a striking association of postoperative TPO dynamics and the development of LD. In particular, HCC patients as well as patients undergoing major resections, who are at high risk of developing postoperative LD, failed to increase TPO production after surgery. The postoperative TPO dynamics might therefore help to identify high-risk patients who could benefit from perioperative TPO substitution. However, the therapeutic potential will have to be assessed in a separate clinical trial.

Besides the therapeutic potential, the use of perioperative TPO levels might enable to identify high-risk patients early after hepatectomy, which might help to modify postoperative treatment. In particular, these patients might benefit from an early additional radiologic evaluation or close monitoring of blood parameters for rapid detection of postoperative LD. This could trigger early postoperative antibiotic therapy if indicated, since patients with LD are known to be more susceptible to infections. Furthermore, the indication for the early use of liver support devices such as the molecular adsorbent recirculating system (MARS) could in part be based on TPO dynamics.

Taken together, perioperative TPO dynamics may not only reflect residual liver function but might also have an important role in hepatic regeneration. Its capacity as an early marker for postoperative LD will have to be prospectively validated. This is of particular interest as perioperative TPO substitution might represent an attractive therapeutic option for patients with underlying liver disease undergoing extensive liver resection.

## Supporting Information

S1 Table Patient Demographics According to Extent of Resection. (DOCX)

S2 Table Patient Demographics According to Postoperative LD. (DOCX)
