# Supplementary material for: Deficiency in Thrombopoietin Induction after Liver Surgery Is Associated with Postoperative Liver Dysfunction
Source: PLoS One. 2015 Jan 22;10(1):e0116985. doi: 10.1371/journal.pone.0116985 (PMC4303418; doi:10.1371/journal.pone.0116985)
Supplement: S1 Table — (DOCX) [file pone.0116985.s001.docx]

| **S1 Table. Patient Demographics According to Extent of Resection** | | |
| --- | --- | --- |
| **Parameter** | **Minor LR (N = 36)**  **50 (72%)**  **19 (28%)**  **46 (67%)**  **23 (33%)**  **46 (67%)**  **7 (10%)**  **20 (30%)**  **6 (8.7%)**  **10 (14.5%)**  **59 (85.5%)**  **33 (47.8%)**  **36 (52.2%)**  **Median (Range)** | **Major LR (N = 33)**  **0.63 (0.22-3.17)**  **102 (40-145)**  **84 (43-418)**  **53 (11-699)**  **31 (17-208)**  **32 (7-196)**  **42 (32.5-49.6)**  **217 (92-470)**  **64 (22-85)**  **Median (Range)**  ***14.5 (5-60)***  ***2 (1-2)*** |
| **Sex** |  |  |
| **Male** | ***30 (83%)*** | ***20 (61%)*** |
| **Female** | ***6 (17%)*** | ***13 (39%)*** |
| **Neoplastic entity** |  |  |
| **mCRC** | ***28 (78%)*** | ***18 (55%)*** |
| **HCC** | ***8 (22%)*** | ***15 (45%)*** |
| **Preoperative CTx** | ***26 (72%)*** | ***20 (61%)*** |
| **Portal Venous Embolization** | **1 (2.8%)** | **6 (18%)** |
| **Pringle maneuver** | **7 (19%)** | **13 (39%)** |
| **RBC intraoperative** | **3 (8%)** | **3 (9%)** |
| **Postoperative LD** |  |  |
| **yes** | ***1 (2.8%)*** | ***9 (27%)*** |
| **no** | ***35 (97%)*** | ***24 (73%)*** |
| **Severe morbidity** | ***4 (11%)*** | ***10 (30%)*** |
| **Preoperative parameters** | **Median (Range)** | **Median (Range)** |
| **TPO pg/ml** | ***43.5 (0-139.5)*** | ***34.6 (0-149.3)*** |
| **PDR %** | ***20.7 (13-36)*** | ***20.9 (7.6-38)*** |
| **R15 %** | ***5.0 (0.4-15.7)*** | ***4.0 (0.3-32)*** |
| **SB mg/dl** | ***0.63 (0.22-2.86)*** | ***0.63 (0.3-3.17)*** |
| **PT %** | ***103 (61-145)*** | ***100 (40-137)*** |
| **ALP U/l** | ***84 (43-165)*** | ***86 (51-418)*** |
| **GGT U/l** | ***43 (16-245)*** | ***68 (11-699)*** |
| **AST U/l** | ***28 (17-175)*** | ***35 (17-208)*** |
| **ALT U/l** | ***24 (11-196)*** | ***37 (7-123)*** |
| **Albumin g/l** | ***42 (33-50)*** | ***42 (34-47)*** |
| **Platelets (x10^3^/µl)** | ***215 (103-470)*** | ***217 (92-431)*** |
| **Age (years)** | ***63 (22-86)*** | ***66 (24-83)*** |
| **Postoperative parameters** | **Median (Range)** | **Median (Range)** |
| **TPO pg/ml** | ***92 (17.7-353.8)*** | ***36.8 (0-243.5)*** |
| **SB mg/dl** | ***1.15 (0.44-4.3)*** | ***1.5 (0.56-4.5)*** |
| **PT %** | ***63 (40-80)*** | ***47 (29-71)*** |
| **ALP U/l** | ***53 (31-105)*** | ***68 (35-286)*** |
| **GGT U/l** | ***55 (13-189)*** | ***64 (6-431)*** |
| **AST U/l** | ***310 (56-1614)*** | ***451 (124-2093)*** |
| **ALT U/l** | ***309 (70-1347)*** | ***404 (86-1769)*** |
| **Albumin g/l** | ***30.8 (20-38)*** | ***28.9 (20-36)*** |
| **Platelets ( x10^3^/µl)** | ***162 (103-378)*** | ***167 (70-444)*** |
| **Postoperative hospitalization** | ***7 (4-32)*** | ***9 (5-90)*** |
| **Intraoperative parameters** | **Median (Range)** | **Median (Range)** |
| **Pringle maneuver min** | ***0 (0-30)*** | ***0 (0-60)*** |
| **RBC intraoperative (Units)** | ***0 (0-2)*** | ***0 (0-2)*** |
| **ALT = alanine aminotransferase, ALP = alkaline phosphatase, AST = aspartate aminotransferase, CTx = chemotherapy, GGT = gamma-glutamyltransferase, HCC = hepatocellular carcinoma, LD = liver dysfunction, LR = liver resection, mCRC = metastatic colorectal cancer, PDR = plasma disappearance rate, PT= prothrombin time, RBC = red blood cells, R15 = retention rate after 15 min, SB = serum bilirubin** | | |
