# Supplementary material for: Deficiency in Thrombopoietin Induction after Liver Surgery Is Associated with Postoperative Liver Dysfunction
Source: PLoS One. 2015 Jan 22;10(1):e0116985. doi: 10.1371/journal.pone.0116985 (PMC4303418; doi:10.1371/journal.pone.0116985)
Supplement: S2 Table — (DOCX) [file pone.0116985.s002.docx]

| **S2 Table. Patient Demographics According to Postoperative LD** | | |
| --- | --- | --- |
| **Parameter** | **No LD (N = 59)**  **50 (72%)**  **19 (28%)**  **46 (67%)**  **23 (33%)**  **46 (67%)**  **7 (10%)**  **20 (30%)**  **6 (8.7%)**  **10 (14.5%)**  **59 (85.5%)**  **33 (47.8%)**  **36 (52.2%)**  **Median (Range)** | **LD (N = 10)**  **0.63 (0.22-3.17)**  **102 (40-145)**  **84 (43-418)**  **53 (11-699)**  **31 (17-208)**  **32 (7-196)**  **42 (32.5-49.6)**  **217 (92-470)**  **64 (22-85)**  **Median (Range)**  ***14.5 (5-60)***  ***2 (1-2)*** |
| **Sex** |  |  |
| **Male** | ***43 (73%)*** | ***7 (70%)*** |
| **Female** | ***16 (27%)*** | ***3 (30%)*** |
| **Neoplastic entity** |  |  |
| **mCRC** | ***43 (73%)*** | ***3 (30%)*** |
| **HCC** | ***16 (27%)*** | ***7 (70%)*** |
| **Preoperative CTx** | ***43 (73%)*** | ***3 (30%)*** |
| **Portal Venous Embolization** | **6 (10%)** | **1 (10%)** |
| **Pringle maneuver** | **15 (25%)** | **5 (50%)** |
| **RBC intraoperative** | **4 (6.8%)** | **2 (20%)** |
| **Hepatic resection** |  |  |
| **Major** | ***24 (41%)*** | ***9 (90%)*** |
| **Minor** | ***35 (59%)*** | ***1 (10%)*** |
| **Severe morbidity** | ***9 (15%)*** | ***5 (50%)*** |
| **Preoperative parameters** | **Median (Range)** | **Median (Range)** |
| **TPO pg/ml** | ***38.9 (0-149)*** | ***56.2 (0-115)*** |
| **PDR %** | ***21 (7.6-38.3)*** | ***17.5 (9.9-26.4)*** |
| **R15 %** | ***4.3 (0.3-32)*** | ***6.6 (1.9-22.7)*** |
| **SB mg/dl** | ***0.62 (0.22-3.17)*** | ***0.93 (0.52-1.63)*** |
| **PT %** | ***102 (45-145)*** | ***100 (40-111)*** |
| **ALP U/l** | ***85 (43-418)*** | ***80 (51-300)*** |
| **GGT U/l** | ***52 (11-490)*** | ***84 (22-699)*** |
| **AST U/l** | ***31 (17-175)*** | ***31 (25-208)*** |
| **ALT U/l** | ***30 (7-196)*** | ***43 (18-120)*** |
| **Albumin g/l** | ***42 (33-50)*** | ***43 (35-47)*** |
| **Platelets (x10^3^/µl)** | ***217 (92-470)*** | ***224 (129-333)*** |
| **Age (years)** | ***64 (22-86)*** | ***67 (51-81)*** |
| **Postoperative parameters** | **Median (Range)** | **Median (Range)** |
| **TPO pg/ml** | ***61.7 (0-354)*** | ***29 (5.3-117)*** |
| **SB mg/dl** | ***1.18 (0.44-3.5)*** | ***2.98 (1.1-4.5)*** |
| **PT %** | ***56 (37-80)*** | ***36 (29-47)*** |
| **ALP U/l** | ***60 (31-286)*** | ***56 (39-176)*** |
| **GGT U/l** | ***59 (6-335)*** | ***81 (41-431)*** |
| **AST U/l** | ***378 (56-2093)*** | ***346 (198-897)*** |
| **ALT U/l** | ***357 (70-1769)*** | ***362 (132-796)*** |
| **Albumin g/l** | ***29 (20-38)*** | ***31 (25-36)*** |
| **Platelets (x10^3^/µl)** | ***167 (70-444)*** | ***143 (77-294)*** |
| **Postoperative hospitalization** | ***8 (4-61)*** | ***12 (5-90)*** |
| **Intraoperative parameters** | **Median (Range)** | **Median (Range)** |
| **Pringle maneuver min** | ***0 (0-30)*** | ***2.5 (0-60)*** |
| **RBC intraoperative (Units)** | ***0 (0-2)*** | ***0 (0-2)*** |
| **ALT = alanine aminotransferase, ALP = alkaline phosphatase, AST = aspartate aminotransferase, CTx = chemotherapy, GGT = gamma-glutamyltransferase, HCC = hepatocellular carcinoma, LD = liver dysfunction, mCRC = metastatic colorectal cancer, PDR = plasma disappearance rate, PT= prothrombin time, RBC = red blood cells, R15 = retention rate after 15 min, SB = serum bilirubin** | | |
